# Slightly better pain relief but more frequently motor blockade with combined nerve block analgesia compared to continuous intraarticular analgesia after total knee arthroplasty

**DOI:** 10.1007/s00167-019-05843-2

**Published:** 2020-02-28

**Authors:** Jörg Lützner, Richard Gehring, Franziska Beyer

**Affiliations:** Department for Orthopaedic and Trauma Surgery, University Medicine Carl Gustav Carus, Fetscherstr. 74, 01307 Dresden, Germany

**Keywords:** Total knee arthroplasty, Total knee replacement, Analgesia, Pain, Function, Quality of life, PROM

## Abstract

**Purpose:**

Pain management after total knee arthroplasty (TKA) is still under debate. Continuous peripheral nerve blocks (PNB) can provide long pain relief but impair muscle function. Continuous intraarticular analgesia could result in longer pain relief than local infiltration analgesia without negative effects on muscle function. This study investigated the efficacy of pain control between PNB’s and continuous intraarticular analgesia after TKA.

**Methods:**

A prospective randomized study on 140 patients undergoing TKA was performed. Patients received either a combination of continuous femoral nerve block, continuous sciatic nerve block and single-shot obturator nerve block (group R) or a local infiltration analgesia and a continuous intraarticular catheter with ropivacaine (group L). Primary outcome was pain measured on a numerical rating scale. Knee function, patient-reported outcome (PRO) and adverse events were assessed until 1 year after surgery.

**Results:**

Pain at rest was lower in group R on the day of surgery (mean NRS 3.0 vs. 4.2) and the morning of postoperative day 1 (mean NRS 3.4 vs. 4.4). Motor blockade longer than postoperative day 3 occurred more often in group R compared to group L (15.3% vs. 1.5%). Pain levels, PRO and satisfaction 3-month and 1-year after surgery were similar.

**Conclusion:**

Continuous PNB’s were slightly more effective in the first 24 h after surgery but were associated more often with motor blockade which should be avoided. It must be balanced if the small amount of better pain relief immediately after surgery justifies the risks associated with motor blockade following PNB’s.

**Level of evidence:**

I.

## Introduction

Despite total knee arthroplasty (TKA) being considered as one of the most painful surgeries, pain control after surgery is still a challenge and there is currently no generally agreed optimal analgesic regimen after TKA [3, 8, 23]. Inadequate pain management can delay rehabilitation, reduce the range of motion (ROM) and may finally result in less favorable outcome and satisfaction [12, 16]. Sufficient postoperative pain relief is, therefore, most important to ensure early mobilization and good results.

Continuous peripheral nerve blocks and periarticular local infiltration analgesia (LIA) are commonly used within multimodal pain management concepts. Both techniques have been shown to provide superior analgesia compared to systemic analgesics alone with less opioid consumption and fewer opioid-related side effects like nausea, vomiting and dizziness [10, 21]. Over more than 2 decades continuous peripheral nerve blocks (PNB) have been preferentially applied for TKA offering probably the best postoperative pain control, especially if femoral nerve block is combined with sciatic nerve block and obturator nerve block [8, 18]. The effect of the combined peripheral nerve blocks has been described as superior to a single continuous femoral nerve block [1, 17, 18]. Continuous PNB can provide long pain relief but impair quadriceps function which may result in prolonged ambulation and increase the risk of falls [7, 20]. Therefore, in the light of enhanced recovery protocols LIA has become the standard procedure for pain management in TKA in the last years. While it has been demonstrated to be very efficient immediately postoperatively [15], the analgesic effect fades after about 1 day. Continuous intraarticular analgesia could result in longer pain relief without impairment of muscle function. Previous studies provided no conclusive results for the use of continuous intraarticular analgesia with infusion pumps. While some studies demonstrated the efficacy of continuous intraarticular analgesia [4, 5, 13] other studies did not find a relevant effect [2, 9, 14, 22, 24].

This study aimed to compare pain control and knee function after TKA with combined continuous nerve blocks or continuous intraarticular analgesia with an infusion pump. We hypothesized similar pain control with both methods but less muscle impairment in continuous intraarticular analgesia.

## Methods

**Fig. 1:**
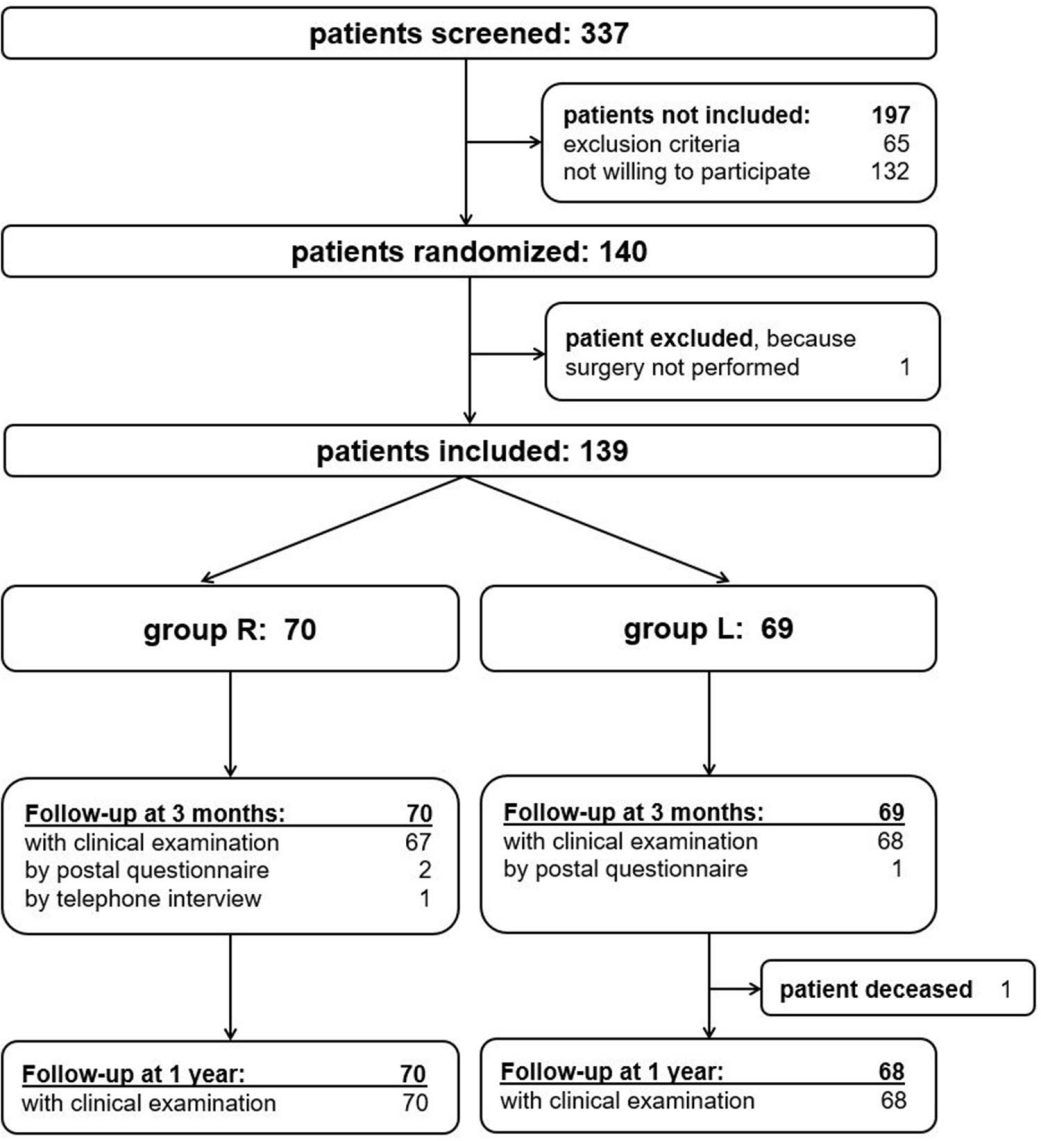
CONSORT flow-chart of study participants

This study was performed as a single-center prospective randomized trial. All patients scheduled for an elective primary unconstrained TKA were screened. Exclusion criteria included allergies against local anaesthetics (none), chronic pain disorders (*n *= 19), dementia (*n *= 2), neuropathic diseases (*n *= 6), other serious illness complicating participation in the study (*n *= 32) or patients which were not able to complete the questionnaires (*n *= 6). After signing informed consent, patients were randomized to receive postoperative regional analgesia with combined continuous peripheral nerve blocks (group R) or a continuous local intraarticular analgesia (group L). Randomisation was performed by using a randomisation list. A total of 140 patients were randomised (Fig. 1). Two patients from group L changed to group R due to preference. One patient from group L did not undergo TKA during the study period. Both groups were similar with regard to age, gender, body mass index (BMI) and duration of the surgery. Despite randomization, there were significantly more patients with severe comorbidities (ASA grade ≥ 3) in group R.

All patients received a cemented, non-constrained, cruciate-retaining condylar TKA (Balansys, Mathys, Bettlach, Switzerland) without patellar resurfacing. The surgery was performed using a tourniquet and a standard medial parapatellar approach.

### Continuous peripheral nerve blocks

Before surgery continuous femoral nerve block was performed under ultrasound guidance (Philips sparq, 12 MHz linear transducer L12-4) using an in-plane needle approach (Contiplex S ultra 18G, BBraun, Melsungen, Germany). 20 ml ropivacaine 0.5% were administered perineurally and a 20G indwelling catheter was advanced 3 cm beyond the needle tip. Correct catheter position was approved by sonographic visualization of perineural spread of 1 ml saline injected via the catheter. Catheters were fixed on skin surface by a sterile fixation kit with a transparent dressing (Perifix cover, BBraun, Melsungen, Germany).

Obturator nerve block was undertaken as single shot with 10 ml ropivacaine 0.5% under ultrasound guidance and in-plane needle advancement (Sonoplex 22G, Pajunk GmbH, Geisingen, Germany).

Patients were then turned into lateral decubitus position and a needle was directed in-plane in a parasacral approach for sciatic nerve block under ultrasound guidance (Philips sparq, 6 MHz curved transducer C6-2) and additional nerve stimulation. 20 ml prilocaine 1.5% were administered and a 20G catheter threatened 3 cm beyond the needle tip. Catheter location approval, skin fixation and dressing were accomplished as described before.

After surgery, a continuous infusion of 6 ml/h of ropivacaine 0.2% was administered via both catheters for postoperative pain relief on the surgical ward.

### Continuous local intraarticular analgesia

**Fig. 2:**
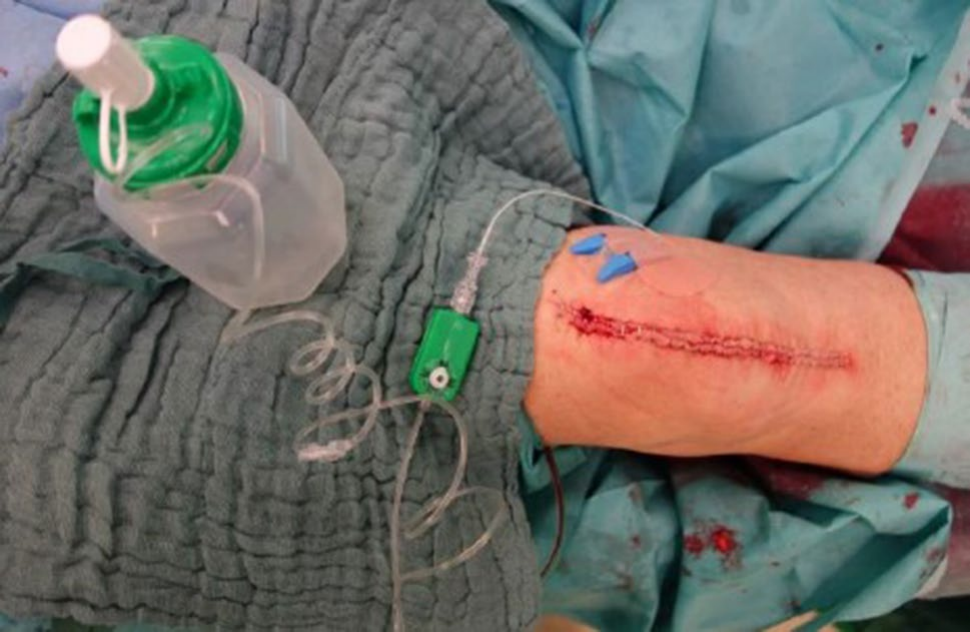
Continuous intraarticular catheter connected via a valve with the ropivacaine pouch

For postoperative analgesia a total of 400 ml Ropivacaine 0.2% were used. After completion of all bone cuts and soft-tissue balancing 20 ml were infiltrated in the posterior capsule and at the posterior cruciate ligament. After cementing the tibial and femoral components additional 30 ml were infiltrated at the collateral ligaments, periosteum, fat pad and around the surgical approach. The remaining 350 ml were filled in an elastic pouch and connected via a valve to a catheter, which was inserted intraarticularly (Fuser Pump, Pajunk, Geisingen, Germany, Fig. 2). The valve was adjusted to administer 8 ml per hour until the pouch was empty which lasted for about 44 h.

### Anesthesia

According to comorbidity and preference of the patient additional sedation (group R only), general or spinal anesthesia were applied for the surgery.

### Postoperative analgesia

In addition to continuous PNB or continuous local intraarticular analgesia all patients received according to the standard hospital pain protocol a basic analgesia with novamine sulfon 1 g each 8 h and additional oral oxycodone 10 mg each 12 h. Further, all patients received piritramid via a patient-controlled intravenous analgesia (PCIA) for the first 3 days after the surgery as a rescue analgesia procedure.

### Outcome measures

Primary outcome was the pain measured with a numerical rating scale (NRS) from 0 (no pain) to 10 (most conceivable pain). The subjective pain perception was recorded by the patient both at rest and during movement in the morning and in the evening daily in a diary. A study nurse took care that the measures were filled in the diary.

The additional necessary pain medication was captured from the patient’s records. The number of rescue pain medications and the total consumption of piritramid was recorded.

Patient characteristics, cut-sew-time, time for preparation before surgery, time after surgery until leaving the OR and total time in the OR were taken from patients records.

Range of motion (ROM) was measured using a goniometer and documented preoperatively, during the hospital stay and during follow-up examinations. The degree of mobilization (in bed, sitting, getting up with help/independently, walking in the room/on the ward and climbing stairs) was documented daily during the hospital stay. All adverse events (including falls and dislocation of the catheters) were recorded.

Additionally, knee function, patient-reported outcome (PRO), health-related quality of life (HrQoL) and satisfaction were assessed using the Knee Society Score (KSS), the Oxford Knee Score (OKS), the EuroQuol questionnaire (EQ-5D) and a visual-analogue scale for satisfaction with the result of the surgery between 0 (worst) and 10 (best).

The study has been performed in compliance with the Helsinki Declaration, has been approved by the local ethics committee (EK 250062015) and registered at www.clinicaltrials.gov (NCT03032133). All patients signed informed consent.

### Statistical analysis

To detect a clinically relevant difference of one grade on the pain NRS with a significance level of 0.05 and a power of 0.8 a total of 64 patients per group were necessary. To account for drop-out a total of 140 patients were included.

The clinical and patient-reported data were assembled into a database and analyzed using SPSS^®^ software (release 25 for Windows^®^). An intention-to-treat (ITT) analysis was performed. Data were reported as means and standard deviation (SD) for continuous values and absolute and relative frequencies for categorical values, respectively. Comparisons between groups were performed by unpaired *t* test for continuous values and Chi-square test for categorical values. Differences between groups were considered to be significant if *p *< 0.05.

## Results

**Fig. 3:**
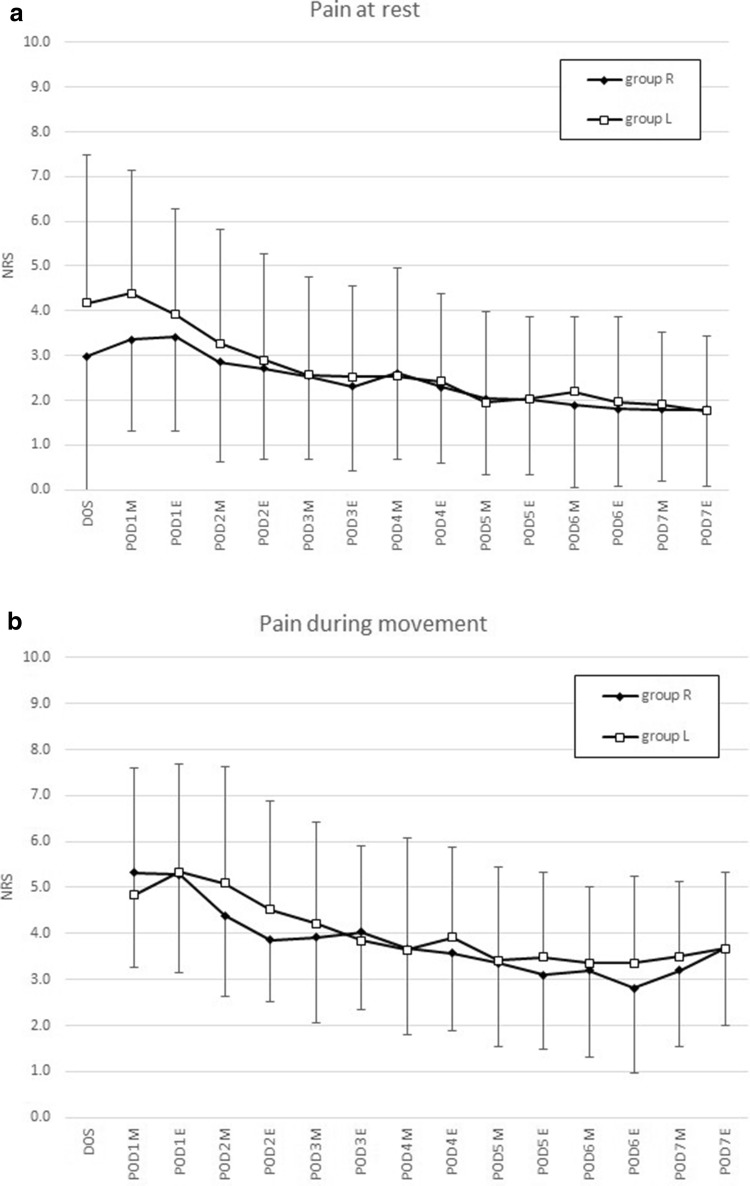
**a** Pain at rest according to the patient diary in both groups given as mean and SD, *DOS* day of surgery, *POD* postoperative day, *M* morning, *E* evening. **b** Pain during movement according to the patient diary in both groups given as mean and SD, *DOS* day of surgery, *POD* postoperative day, *M* morning, *E* evening

Pain at rest was significantly lower in group R on the day of surgery (mean NRS 3.0 vs. 4.2, *p* = 0.037) and the morning of postoperative day 1 (mean NRS 3.4 vs. 4.4, *p* = 0.015). There were no differences later on, and no differences at all during movement (Fig. 3). Pain levels after 3 months (mean NRS 1.8 group R vs. 1.5 group L) and 1 year were similar (mean NRS 1.0 in both groups). Patients in group R needed significantly less Piritramid via PCIA on postoperative day 1 (22.7 mg vs. 35.3 mg, *p* = 0.001) and day 2 (15.2 mg vs. 24.1 mg, *p* = 0.009).

Peripheral nerve blocks needed significantly more preparation time before surgery but less total OR time (Table 1). Motor blockade longer than postoperative day 3 occurred in 15.3% in group R compared to 1.5% in group L (*p *= 0.01). In two patients (group R) incomplete femoral nerve palsy with resulting quadriceps weakness persisted after discharge but disappeared within 3-month. Independent walking on the ward was possible on postoperative day 2 in 44% in group R and 56% in group L (*p* 0.037). Catheter-related problems (dislocation, leakage) occurred significantly (*p* = 0.017) more often with peripheral nerve catheters (11.4%) than with the intraarticular catheters (1.4%).

**Table 1:** Characteristics of study participants given as mean (SD) or absolute (relative) frequencies

	R	L	*p* value
Age at surgery (years)	68.0 (9.4)	68.7 (8.1)	n.s.
BMI (kg/m^2^)	31.7 (5.4)	30.1 (5.2)	n.s.
Gender (% female)	40 (57.1%)	34 (49.3%)	n.s.
Comorbidities (≥ ASA 3)	38 (54.3%)	22 (31.9%)	0.008
Cut-sew-time (min)	76.8 (12.6)	78.4 (11.1)	n.s.
Preparation time before surgery (min)	56.6 (26.9)	19.7 (12.3)	< 0.001
Time after surgery in the OR (min)	13.5 (5.1)	16.1 (6.5)	0.011
Total time in the OR (min)	118.1 (16.4)	124.8 (15.1)	0.013

Knee function, PRO and HrQoL demonstrated no significant differences between both groups at any follow-up and both groups reached a high satisfaction score (Table 2).

**Table 2:** Knee function and patient-reported outcome measures given as mean (SD)

	R	L	*p* value
ROM (in degrees)			
Preop	96.4 (16.1)	97.4 (16.5)	n.s.
3 month	101.1 (12.3)	101.5 (11.8)	n.s.
1 year	109.6 (12.6)	110.4 (10.8)	n.s.
Improvement	13.2 (16.6)	12.9 (17.6)	n.s.
Knee Score (max. 100 points)			
Preop	38.5 (15.2)	40.4 (16.2)	n.s.
3 month	79.3 (14.2)	81.4 (11.8)	n.s.
1 year	86.9 (12.0)	88.3 (9.4)	n.s.
Improvement	48.4 (21.3)	47.6 (16.7)	n.s.
Function Score (max. 100 points)			
Preop	53.7 (12.3)	52.7 (10.4)	n.s.
3 month	64.5 (16.9)	68.0 (19.2)	n.s.
1 year	74.6 (17.5)	78.8 (16.2)	n.s.
Improvement	20.9 (16.3)	26.0 (16.0)	n.s.
Oxford Knee Score (max. 48 points)			
Preop	23.5 (6.1)	24.3 (5.3)	n.s.
3 month	32.9 (8.1)	33.7 (6.7)	n.s.
1 year	37.8 (7.1)	38.4 (6.7)	n.s.
Improvement	14.3 (7.9)	14.1 (7.6)	n.s.
EQ-5D Index (max. 1.0)			
Preop	0.625 (0.266)	0.689 (0.193)	n.s.
3 month	0.875 (0.119)	0.868 (0.158)	n.s.
1 year	0.872 (0.173)	0.884 (0.172)	n.s.
Improvement	0.240 (0.267)	0.196 (0.261)	n.s.
Satisfaction at 1 year follow-up (max. 10)	8.5 (1.4)	8.8 (1.4)	n.s.

Adverse events were not significantly different between both groups. There were two periprosthetic infections in group L, which could be successfully treated with debridement, antibiotics and implant retention (DAIR). Furthermore, there were two superficial wound revisions (group L), one hematoma (group L), one wound dehiscence after a fall (group R) and one removal of a fixed drainage (group R). In six patients with restricted ROM a manipulation under anesthesia was performed, four in group R and two in group L.

## Discussion

The most important finding of this study was a better pain relief of one NRS grade of continuous peripheral nerve blocks compared to continuous intraarticular analgesia within the first 24 h. This had no influence on knee function, patient-reported outcome or patient satisfaction. The better pain relief was associated with a higher rate of motor blockade after surgery impairing early ambulation on the ward.

The positive effect of continuous intraarticular injection of morphine and ropivacaine on knee flexion and hospital stay was described by Rasmussen et al. [13]. Further studies demonstrated the positive effect in pain reduction of continuous intraarticular application of local anesthetics compared to placebo [4, 5]. Other studies did not find relevant effects of continuous intraarticular local anesthetics compared to placebo on pain, analgesic consumption or function [2, 22]. Reinhardt et al. [14] demonstrated higher pain scores in continuous intraarticular ropivacaine infusion compared to continuous epidural analgesia plus femoral nerve block but less knee-buckling and faster ambulation. Zinkus et al. [24] realized less opioid use, lower motor blockade and better knee function in continuous intraarticular analgesia compared to the continuous femoral nerve block. In all these studies, different pain protocols were used with different nerve blocks, different local anesthetics and different additional pain medication making it difficult to compare the studies directly.

In the present study, a relatively low-dose LIA with only 50 ml ropivacaine 0.2% was used and the intraarticular catheter probably needs some time until a sufficient amount of local anesthetics has infiltrated the knee to be effective. The PNB’s have been administered about 1 h before skin incision and had already full effect at the end of the surgery which might explain the superior pain relief immediately after surgery. It might be favorably to use a higher dose of LIA to have an immediate effect after surgery. However, compared to other studies the pain relief of the intraarticular analgesia was similar to pain NRS grade 4 on the day of surgery and postoperative day 1 [9, 14]. Beyond 24 h, continuous PNB’s did not result in superior pain relief. A high incidence of catheter dislocations of PNB’s has been reported with the consequence of less effective pain relief. Terkawi al. [18] found that most failures of PNB were related to catheter dislocations. In the present study in 11.5% of the PNB dislocations or leakage of the catheters were recognized.

Consistent with other studies PNB’s were associated with more motor blockade and slower mobilization which is a disadvantage as early mobilization is very important after TKA. It has been demonstrated that early mobilization reduces adverse events after TKA [6]. Falls as a result of motor blockade after femoral nerve catheter have been described in nearly 3% [11] with possible significant complications such as wound dehiscence, infection or even periprosthetic fracture which need to be prevented. Therefore, other pain management methods have been searched which do not affect the muscle strength after surgery. LIA combined with continuous intraarticular analgesia or adductor canal block [19] might be effective methods to control pain without affecting muscle function after TKA.

This study has some limitations. Not all patients received randomized treatment. Therefore, the ITT analysis does not exactly reflect the true treatment. However, the number of patients who changed groups was small and we performed an additional “as-treated” analysis, which demonstrated no different results. Despite randomization, patients in group R had more comorbidities, which might have negatively affected mobilization. The LIA used in this study was relatively low-dose and a higher amount of local anesthetics might be necessary for immediate postoperative pain relief. Finally, pain is a perception and cannot be objectified. Measurement is therefore highly subjective and the used NRS scale only an approximation. However, this is an inherent limitation of all studies focusing on pain.

## Conclusion

It must be balanced if the small amount of better pain relief immediately after surgery justifies the risks associated with motor blockade following PNB’s. The combination of LIA and continuous intraarticular analgesia might be an adequate alternative.

## Abbreviations

BMI: Body mass index
DOS: Day of surgery
E: Evening
EQ-5D: EuroQuol questionnaire
HrQoL: Health-related quality of life
ITT: Intention-to-treat
KSS: Knee Society Score
LIA: Local infiltration analgesia
M: Morning
NRS: Numerical rating scale
OKS: Oxford Knee Score
OR: Operating room
PCIA: Patient-controlled intravenous analgesia
PNB: Peripheral nerve blocks
POD: Postoperative day
PRO: Patient reported outcome
ROM: Range of motion
SD: Standard deviation
TKA: Total knee arthroplasty
